# TiO2 NPs induced hepatic injury in mammals: a mechanistic approach

**DOI:** 10.1186/1755-8166-7-S1-P82

**Published:** 2014-01-21

**Authors:** Ritesh K Shukla, Ashutosh Kumar, NV Srikanth Vallabani, Sanjay Singh, Alok Dhawan

**Affiliations:** 1 Institute of Life Sciences, Ahmedabad University, Opposite University Bus Stand, University Road, Navrangpura, Ahmedabad, Gujarat, India

## Background

The rapid advancement in nanotechnology has increased the production of metal oxide nanoparticles (NPs) especially TiO_2_ for consumer and industrial products. This has also increased the likelihood for their exposure to human. TiO_2_ NPs exposure to humans can occur through different routes, but will finally reach to liver through the circulatory system. Hence, the present study was planned to assess the effects of TiO_2_ NPs in mammalian liver and their possible mechanism.

## Materials and methods

TiO_2_ NPs were characterized by transmission electron microscopy (TEM) and dynamic light scattering (DLS). Genotoxicity assessment of TiO_2_ NPs was carried out by fpg-modified Comet assay both in in vitro (HepG2 cells) and in vivo (mice liver). Additionally, to understand the mechanism of hepatotoxicity, biochemical parameters, oxidative stress markers, reactive oxygen species (ROS), and expression profile of different stress proteins, tumour suppressor apoptotic/antiapoptotic proteins were investigated.

## Results

TEM measurements and DLS analysis showed that TiO_2_ NPs were in nano size regime, stable and mono-dispersed in different exposure vehicles, making them suitable for in vitro and in vivo toxicity studies. Our data from in vitro and in vivo study exhibited that TiO_2_ NPs induced significant (p<0.05) oxidative DNA damage assessed by the fpg-Comet assay. This could be attributed to a concentration-dependent significant (p<0.05) increase of ROS generation as evident from the enhanced fluorescence intensity of DCFDA dye. A significant alteration in the level of different hepatic enzymes in TiO_2_ NPs treated mice was also observed.

Furthermore, immunoblot analysis revealed a significant increase in the expression profile of Hsp60, Hsp70, p53, BAX, Cyto-c, Apaf-1, caspase-9 and caspase-3 protein and a concomitant decrease in the level of antiapoptotic protein Bcl-2. Our data demonstrate the role of mitochondrial intrinsic pathway for TiO_2_ NP induced apoptosis in liver cells.

## Conclusion

The present study using fpg-modified Comet assay, blood biochemical parameters, oxidative stress markers and immunoblot analysis confirmed that oxidative stress induced by TiO_2_ NPs trigger the DNA damage, which consequently initiates the expression of apoptotic proteins resulting in hepatic injury. Hence the use of such nanoparticles should be carefully monitored.

